# Working memory load and distraction: dissociable effects of visual maintenance and cognitive control

**DOI:** 10.3758/s13414-014-0742-z

**Published:** 2014-08-02

**Authors:** Nikos Konstantinou, Eleanor Beal, Jean-Remi King, Nilli Lavie

**Affiliations:** 1 Center for Applied Neuroscience, University of Cyprus, 75 Kallipoleos, P.O. Box 20537, CY 1678 Nicosia, Cyprus; 2 Institute of Cognitive Neuroscience, University College London, London, UK; 3 Cognitive Neuroimaging Unit, Institut National de la Santé et de la Recherche Médicale, U992, 91191 Gif/Yvette, France; 4 NeuroSpin Center, Institute of BioImaging Commissariat à l’Energie Atomique, 91191 Gif/Yvette, France; 5 Institut du Cerveau et de la Moelle Épinière Research Center, Institut National de la Santé et de la Recherche Médicale, U975, Paris, France

**Keywords:** Memory: Visual working and short-term memory, Attention: Selective attention and memory

## Abstract

We establish a new dissociation between the roles of working memory (WM) cognitive control and visual maintenance in selective attention as measured by the efficiency of distractor rejection. The extent to which focused selective attention can prevent distraction has been shown to critically depend on the level and type of load involved in the task. High perceptual load that consumes perceptual capacity leads to reduced distractor processing, whereas high WM load that reduces WM ability to exert priority-based executive cognitive control over the task results in increased distractor processing (e.g., Lavie, *Trends in Cognitive Sciences, 9*(2), 75–82, 2005). WM also serves to maintain task-relevant visual representations, and such visual maintenance is known to recruit the same sensory cortices as those involved in perception (e.g., Pasternak & Greenlee, *Nature Reviews Neuroscience, 6*(2), 97–107, 2005). These findings led us to hypothesize that loading WM with visual maintenance would reduce visual capacity involved in perception, thus resulting in reduced distractor processing—similar to perceptual load and opposite to WM cognitive control load. Distractor processing was assessed in a response competition task, presented during the memory interval (or during encoding; Experiment 1a) of a WM task. Loading visual maintenance or encoding by increased set size for a memory sample of shapes, colors, and locations led to reduced distractor response competition effects. In contrast, loading WM cognitive control with verbal rehearsal of a random letter set led to increased distractor effects. These findings confirm load theory predictions and provide a novel functional distinction between the roles of WM maintenance and cognitive control in selective attention.

The extent to which selective focused attention allows people to successfully ignore irrelevant distractions is central to our understanding of attention and cognitive control. It is now well established that the ability to ignore irrelevant distractions is not determined just by the intention to be focused or by the separability of the target and distractor stimuli, but also by the level and type of processing load involved in the current task (for reviews, see, e.g., Lavie, 1995, 2005, 2010; Lavie & Dalton, 2013; Lavie & Tsal, 1994).

The role of processing load in distractor processing has been proposed in Lavie’s load theory (e.g., Lavie, 1995; Lavie, Hirst, De Fockert, & Viding, 2004), which applied a capacity approach to selective attention, while taking into account the role of priority-based working memory (WM) control (for reviews, see Lavie, 2000, 2012). According to this approach, perception has limited capacity, but capacity has to be allocated to the full to the processing of all stimuli within these limits. Cognitive control over information processing is limited to prioritization of relevant over irrelevant information. These processing priorities are actively maintained in WM, so that capacity is allocated with a higher priority to the relevant information. However, if processing the relevant information does not take up all available capacity, any remaining capacity is allocated involuntarily to the processing of irrelevant information as well (in a simultaneous parallel manner). It follows, then, that the level of perceptual load in the task processing plays a critical role. Task conditions of low perceptual load—for example, detection of a single item or of one that pops out from among dissimilar items—result in distractor processing even if people attempt to ignore irrelevant distractors. Task conditions of higher perceptual load—for example, increased number of items or more complex perceptual processing demands, such as discriminating conjunctions of features (e.g., Lavie, 1995)—result in reduced processing of irrelevant distractors, simply due to reduced availability of perceptual resources.

Load on WM cognitive control functions also plays an important role. Conditions of high WM load that reduce its availability to exert priority-based control over the task result in increased processing of irrelevant distractors (due to the reduced distinction between relevant and irrelevant information). Thus, WM load has the opposite effect on distractor processing to that of perceptual load (e.g., Lavie, 2005; Lavie et al., 2004).

However, WM is a complex system consisting of not only executive cognitive control functions (typically revealed in tasks that load verbal WM), but also visual maintenance functions (shown in visual and spatial WM tasks). These functions are known to differentially recruit frontal (executive control) and posterior (visual maintenance) visual cortices (for reviews, see Repovs & Baddeley, 2006; Smith & Jonides, 1999).

The present research investigates how these two important functions of WM—executive cognitive control and visual maintenance—can be dissociated through the opposite effects of load on selective attention. As we outline above, load theory predicts that load on cognitive control WM functions leads to increased distraction. What should load theory predict for the effects of load on visual memory maintenance? Recent studies have demonstrated that the sensory visual cortex (including the primary visual cortex, area V1) is recruited during visual maintenance (for a review, see Pasternak & Greenlee, 2005; for recent demonstrations, see Ester, Serences, & Awh, 2009; Harrison & Tong, 2009; Malecki, Stallforth, Heipertz, Lavie, & Duzel, 2009; Munneke, Heslenfeld, & Theeuwes, 2010; Serences, Ester, Vogel, & Awh, 2009). Considering these findings within the framework of load theory led us to hypothesize that loading visual short-term memory (VSTM) maintenance would increase demand for the sensory processing capacity that is involved in visual perception, thus leading to reduced distractor processing and enhancing focused selective attention task, much like increasing perceptual load does (e.g., Lavie, 1995).

Preliminary support for the effects of VSTM load on visual perception comes from our recent demonstrations that both detection sensitivity and retinotopic cortex responses—measured for a contrast increment during a memory task delay—are reduced by increased VSTM load (Konstantinou, Bahrami, Rees, & Lavie, 2012; Konstantinou & Lavie, 2013). However, the effects of VSTM load on selective attention, and specifically on distractor interference, have not yet been addressed. Thus, we set out to establish the effects of VSTM load on selective attention, both at maintenance and at encoding (Experiment 1), and compared these with the effects of WM cognitive control load (Experiment 2). In both experiments, we used the response competition task to assess the extent to which people could efficiently use selective attention to avoid distractor interference.

## Experiment 1

**Fig. 1:**
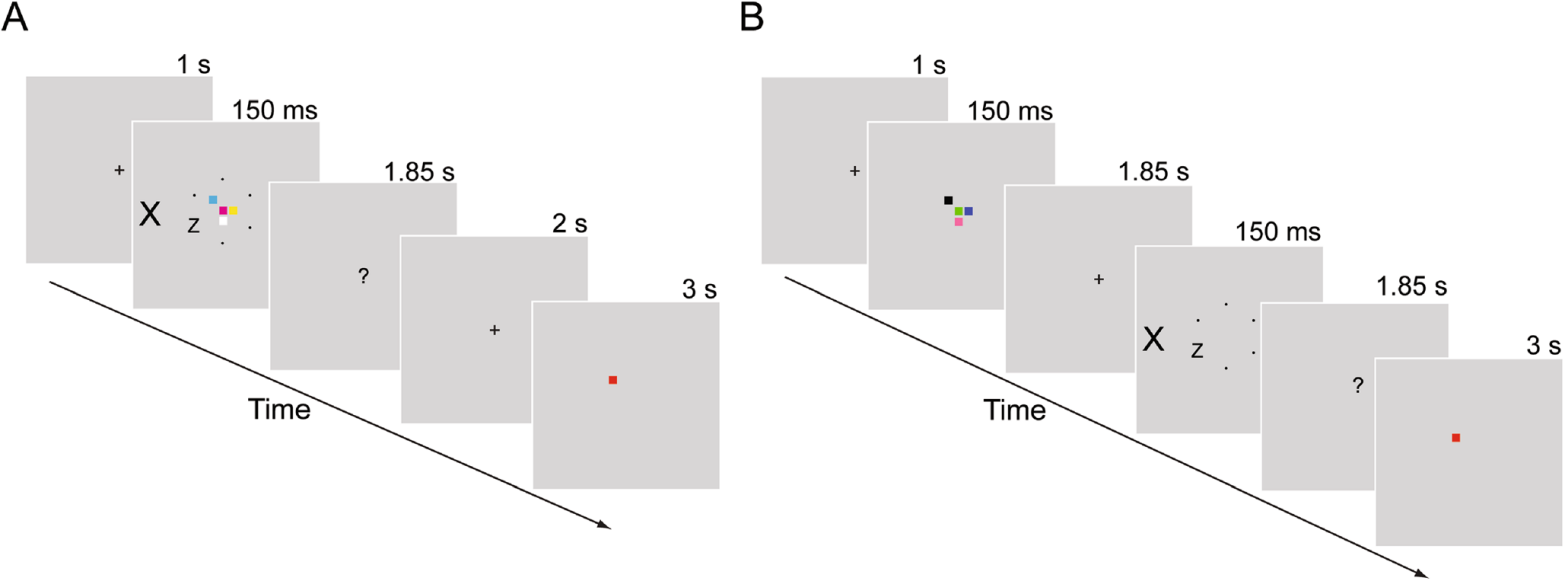
Experiment 1: Example trial sequence in the high-load conditions in **a** Experiment 1a (visual short-term memory [VSTM] encoding load) and **b** Experiment 1b (VSTM maintenance load). In the low-load conditions, the memory set included only one square. Note that the only difference between sequences A and B is in the presentation of the response competition task during either memory encoding or maintenance. An incongruent response competition condition is shown (in the congruent condition, the target and distractor letters were the same). The correct memory probe response here is “different.” Display durations appear above each display. Stimuli are not drawn to scale

Figure 1 shows the stimuli and trial sequence and durations. Participants performed a VSTM task that required matching of a memory probe color and location to those in a memory sample of colored squares (e.g., Luck & Vogel, 1997; Todd & Marois, 2004). Load was manipulated by varying the number of items in the memory set array (one in the low-load and four in the high-load conditions). During this task, participants also engaged in a response competition task (e.g., Eriksen & Eriksen, 1974) that required speeded responses to a target letter in the presence of a congruent distractor (same as target letter—e.g., distractor “X” when the target was an “X”) or an incongruent distractor (e.g., distractor “Z” when the target was an “X”) presented in the periphery. Longer response times (RTs) to the target letter in the incongruent versus the congruent condition indicated a failure to ignore the distractor letter.

The response competition task was presented either during encoding of the VSTM task stimuli (Experiment 1a) or during the delay period of the VSTM task (Experiment 1b; see Fig. 1). In this way, the increased memory sample set size increased either load on encoding into VSTM (a process akin to perceptual load, because a greater number of stimuli needed to be perceived with a higher memory set size) or VSTM maintenance load (because a greater number of stimuli needed to be maintained in VSTM) during the processing of the response competition task stimuli.^1^ We hypothesized that loading sensory visual representation capacity by manipulating either VSTM maintenance or encoding load would lead to reduced distractor processing (see Note 1).

## Experiment 1a

### Method

#### Participants

Seventeen participants (age, 20–29 years; 8 women) took part in Experiment 1a. One outlier participant with response competition RTs more than 2 *SD*s away from the mean was replaced with a new participant. All participants in this and subsequent experiments had normal or corrected-to-normal vision without color blindness; they were recruited from the UCL participant pool and gave informed consent that was approved by the local ethics committee.

#### Stimuli and procedure

The experiment (and all subsequent experiments) was controlled using the Cogent Toolbox (http://www.vislab.ucl.ac.uk/cogent.php) for MATLAB (MathWorks, Inc.) on a Dell PC running Microsoft Windows XP attached to a Sony 15-in. CRT monitor (90-Hz refresh rate). A viewing distance of 60 cm was maintained with a chinrest.

As is shown in Fig. 1, trials started with a fixation cross, followed by a memory set of one (low load) or four (high load) colored squares (0.38° × 0.38°) randomly placed on a 3 × 3 grid (1.38° × 1.38°) centered at fixation. Each square was of a different color, chosen randomly from black (<0.01 cd/m^2^), blue (*x* = .15, *y* = .07; 29.05 cd/m^2^), cyan (*x* = .20, *y* = .27; 69 cd/m^2^), green (*x* = .27, *y* = .59; 65.84 cd/m^2^), magenta (*x* = .28, *y* = .14; 48.20 cd/m^2^), pink (*x* = .32, *y* = .30; 69.14 cd/m^2^), red (*x* = .62, *y* = .33; 39.56 cd/m^2^), white (77 cd/m^2^), and yellow (*x* = .40, *y* = .49; 73.61 cd/m^2^). Display backgrounds were mid-gray (*x* = .27, *y* = .29; 64.11 cd/m^2^). Participants were instructed to maintain the memory set squares throughout the retention interval and respond whether a memory probe square appearing at the end of the interval was the same color as or a different color from the color of the square at the same location in the memory set.

For the response competition task, a circle (2° in radius) of small black dots containing one of two target letters (“X” or “Z,” subtending 0.6° × 0.4°) was presented around the memory set items. Participants searched for the target letter among the small black dots in the empty locations. The target letter was equally likely to appear on any of the six positions of the circle. A distractor letter (subtending 1° × 0.6°) that was equally likely to be congruent (e.g., distractor “X” when the target was “X”) or incongruent (distractor “Z” when the target was “X”) with the target letter appeared 3.5° to the left or to the right of the fixation point.

A display with “?” at the center appeared after the stimulus display for 1.85 s, during which participants responded to the target letter by pressing 0 for “X” or 2 for “Z” using the numerical keypad. An auditory tone (“beep”) was used as feedback for incorrect responses. A blank screen appeared next for 2 s, comprising a VSTM delay interval of 4 s followed by the memory probe display for 3 s (Fig. 1).

The memory probe appeared next, comprising a single square presented in one of the occupied memory set positions. Participants pressed “S” to indicate that the square matched one of the memory set squares in both color and position or “A” to indicate “different.” The memory probe was a match on half of the trials and had a different color on the other half. In the VSTM task, responses were not speeded, and no response feedback was given.

The condition of load was blocked in an ABBABAAB design counterbalanced across participants. Each participant completed a total of eight blocks of 48 trials each (four low-load and four high-load blocks). Prior to the experiment, participants completed two practice blocks of 16 trials each (one low load and one high load, in the same order as the first two blocks of the experiment).

### Results and discussion

VSTM task accuracy rates were significantly lower in the high-load (*M* = 65 %, *SD* = 9 %) than in the low-load (*M* = 87 %, *SD* = 15 %) condition, *t*(16) = 6.36, *p* < .001, *d* = 1.34. The estimated amount of information maintained in VSTM using Cowan’s *K* (Cowan et al., 2005; *K* = *N* [hit rate – false alarm rate], where *K* is the memory estimate and *N* is the number of items presented in the memory set) increased significantly from the low (*K* = 0.73, *SD* = 0.28) to the high (*K* = 1.13, *SD* = 0.68) VSTM load condition, *t*(16) = 2.08, *p* = .012, *d* = 0.72. Thus, the VSTM load manipulation used was effective in taxing VSTM capacity.

Our main hypothesis concerned the effects of VSTM encoding load on distractor interference. Only data from correct VSTM task responses were entered into analyses of the response competition task, and trials with incorrect responses in the response competition task were removed from the RT analyses.

**Fig. 2:**
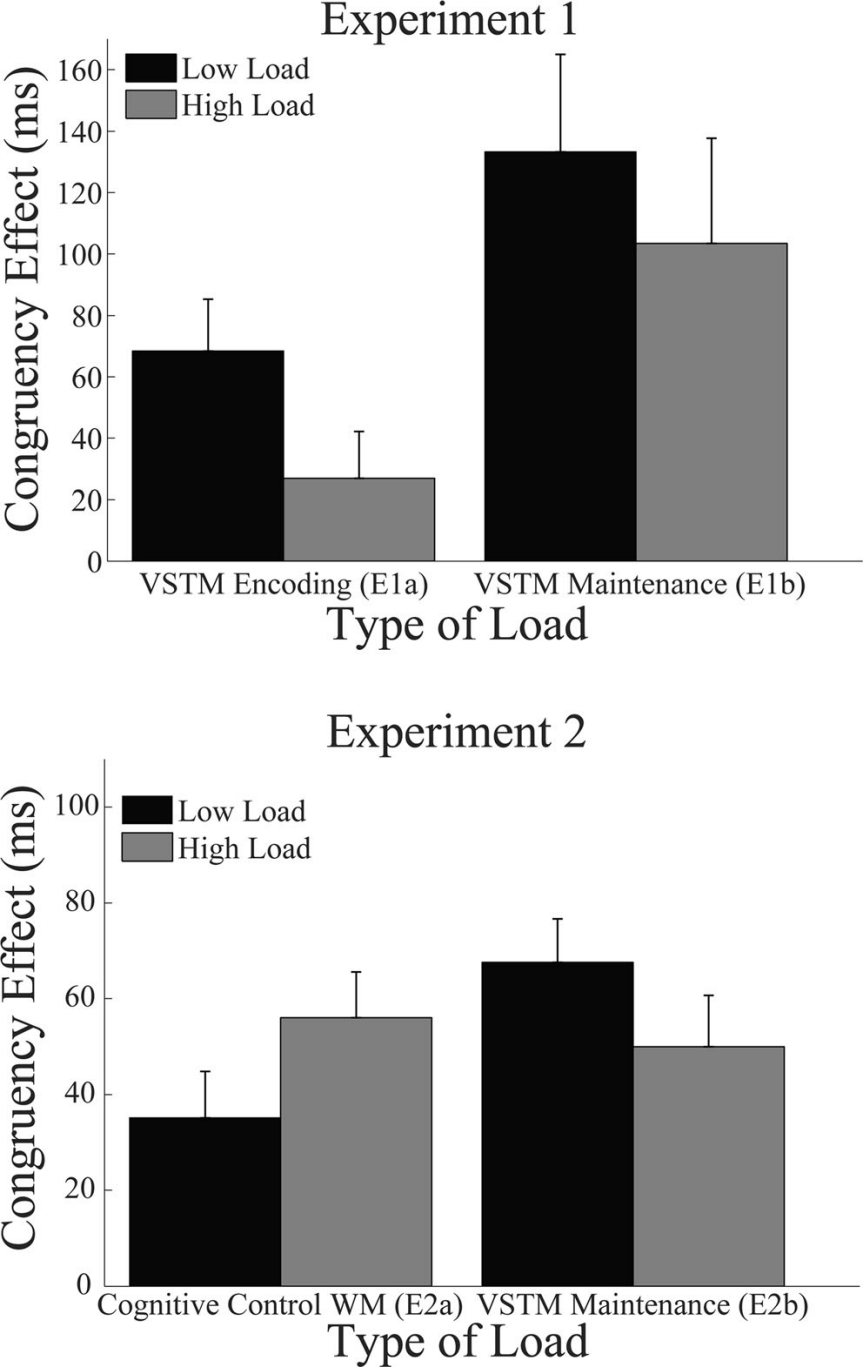
Results of Experiments 1 and 2. Mean congruency effect (incongruent minus congruent condition response time [RT]) as a function of load level and type. RT data are shown only for correct responses on both the memory and response competition tasks. Error bars represent the standard errors of the means

A two-way repeated measures ANOVA on the RTs as a function of load (low, high) and distractor congruency (congruent, incongruent) revealed a main effect of distractor congruency, *F*(1, 16) = 11.67, *p* = .004, *η* ^2^ = .42, indicating that RTs were longer in the presence of incongruent, as compared with congruent, distractors (see Fig. 2). The mean RT was 499 ms (*SD* = 155) in the low-load condition and 474 ms (*SD* = 172) in the high-load condition, and these were not significantly different, *F*(1, 16) = 4.25, *p* = .06, *η* ^2^ = .21 (see Note 1). Critically, there was a significant interaction between load and congruency on RTs, *F*(1, 16) = 6.62, *p* = .02, *η* ^2^ = .29. As is shown in Fig. 2, this interaction reflected a reduced distractor congruency effect with higher VSTM encoding load, as we predicted.^2^

Further inspection of the data revealed that the effect of load on distractor congruency was mostly due to higher load reducing interference by the incongruent distractor (low load, *M* = 534 ms, *SD* = 169 ms; high load, *M* = 487 ms, *SD* = 179 ms), *t*(16) = 2.63, *p* = .018, *d* = 0.27, rather than changing a potentially facilitatory effect by the congruent distractor (low load, *M* = 466 ms, *SD* = 148 ms; high load, *M* = 460 ms, *SD* = 169 ms), *t* < 1. This finding is in line with the traditional notion that distractor congruency effects reflect interference, rather than facilitation (for discussions, see Lavie, 1995; Santee & Egeth, 1982), and load has reduced this interference.

A similar two-way repeated measures ANOVA on the arcsine-transformed accuracy rates of the response competition task revealed a main effect of distractor congruency, *F*(1, 16) = 22.49, *p* < .001, *η* ^2^ = .58, indicating that accuracy rates were lower in the presence of incongruent (*M* = 1.17, *SD* = 0.15), as compared with congruent (*M* = 1.33, *SD* = 0.12), distractors. There was no main effect of load (low load, *M* = 1.24, *SD* = 0.11; high load, *M* = 1.26, *SD* = 0.13), *F* < 1, and no interaction, *F*(1, 16) = 2.595, *p* = .13, *η* ^2^ = .14.

## Experiment 1b

### Method

#### Participants

Twenty-two new participants (ages, 18–51 years; 14 women) took part in Experiment 1b.

#### Stimuli and procedure

The VSTM and selective attention tasks were the same as in Experiment 1a, except that the selective attention task now appeared following a blank screen presented for 1.85 s after the memory set display offset (see Fig. 1b).

### Results and discussion

Accuracy rates in the VSTM task were significantly lower in the high (*M* = 70 %, *SD* = 12 %) than in the low (*M* = 91 %, *SD* = 9 %) VSTM load condition, *t*(21) = 9.61, *p* < .001, *d* = 1.39. The memory estimates (Cowan’s *K*) were significantly increased from the low (*K* = 0.81, *SD* = 0.19) to the high (*K* = 1.53, *SD* = 0.96) VSTM load condition, *t*(21) = 3.96, *p* = .001, *d* = 0.93. These results confirm that VSTM load taxed VSTM capacity as before.

We note that overall, the memory estimates we report (as well as those reported in Experiment 1a) appear lower, as compared with previous studies that have employed a similar VSTM task (e.g., Todd & Marois, 2004) but have used a single-task paradigm. It is likely that our use of a dual-task paradigm has caused some disruption to the memory performance (with our added response competition task serving as a memory distractor task; see, e.g., Awh, Vogel, & Oh, 2006; Clapp, Rubens, & Gazzaley, 2009; Rutman, Clapp, Chadick, & Gazzaley, 2009; Sakai & Passingham, 2004; Yoon, Curtis, & D’Esposito, 2006). Importantly, any memory disruption caused by our use of a dual-task paradigm applied across the load conditions and did not impair our task sensitivity to reveal the specific effects of load on distraction.

A mixed-model ANOVA comparison of the VSTM task arcsine-transformed accuracy rates and the memory estimates between Experiments 1a and 1b as a function of load revealed no interaction [accuracy rates, *F* < 1; memory estimates, *F*(1, 39) = 1.81, *p* = .19, *η* ^2^ = .04] and no main effect for experiment [accuracy rates, *F*(1, 38) = 1.81, *p* = .19, *η* ^2^ = .05; memory estimates, *F*(1, 38) = 2.38, *p* = .13, *η* ^2^ = .06]. Thus, the increase in demands on VSTM capacity with increased memory set size was equivalent between these experiments, as would be expected given the use of the same task.

For the response competition task, a two-way repeated measures ANOVA as a function of VSTM load (low, high) and distractor congruency (congruent, incongruent) revealed a main effect of distractor congruency on RTs, *F*(1, 22) = 32.34, *p* < .001, *η* ^2^ = .60, indicating that RTs were longer in the presence of incongruent, as compared with congruent, distractors. No main effects of load on RTs were found (low load, *M* = 754 ms, *SD* = 223 ms; high load, *M* = 744 ms, *SD* = 222 ms), *F* < 1. Critically for our hypothesis concerning the effects of VSTM maintenance load on distraction, there was a significant interaction between load and congruency on RTs, *F*(1, 22) = 5.36, *p* = .03, *η* ^2^ = .20, reflecting a reduced distractor congruency effect on RTs with higher VSTM maintenance load, as we predicted (see Fig. 2). As was the case in Experiment 1a, the effect of load on distractor congruency was driven mostly by high load reducing interference by the incongruent distractor (low load, *M* = 822 ms, *SD* = 248 ms; high load, *M* = 797 ms, *SD* = 251 ms), *t*(22) = 1.66, *p* = .11, *d* = 0.10), rather than a facilitatory effect by the congruent distractor (low load, *M* = 688 ms, *SD* = 208 ms; high load, *M* = 693 ms, *SD* = 205 ms), *t* < 1.

A similar two-way repeated measures ANOVA on the arcsine-transformed accuracy rates revealed a main effect of distractor congruency, *F*(1, 22) = 7.06, *p* = .01, *η* ^2^ = .24, indicating that accuracy rates were lower in the presence of incongruent, as compared with congruent, distractors. There was no main effect of load (low load, *M* = 1.33, *SD* = 0.12; high load, *M* = 1.31, *SD* = 0.12) and no interaction (both *F*s < 1), similar to Experiment 1b.

Mixed-model ANOVAs were performed on the distractor congruency effects (expressed as the difference between incongruent and congruent distractor conditions) on RTs and on arcsine-transformed accuracy rates with level of load (low, high) as the within-subjects factor and experiment (Experiment 1a, Experiment 1b) as the between-subjects factor. These analyses revealed a significant effect for experiment on RTs, *F*(1, 38) = 6.82, *p* = .01, *η* ^2^ = .15, indicating a greater congruency effect in Experiment 1b (*M* = 118 ms, *SD* = 100 ms) than in Experiment 1a (*M* = 48 ms, *SD* = 57 ms). This might be due to the use of different participant groups with longer RTs overall in Experiment 1b.^3^

There was no main effect of experiment on accuracy rates, *F*(1, 38) = 2.28, *p* = .14, *η* ^2^ = .06. Critically, there were no interactions between the level of load and type of load (at encoding in Experiment 1a vs. maintenance in Experiment 1b) for either RTs or accuracy rates (both *F*s < 1), suggesting a similar effect of VSTM load at encoding (Experiment 1a) and at maintenance (Experiment 1b).

## Experiment 2

**Fig. 3:**
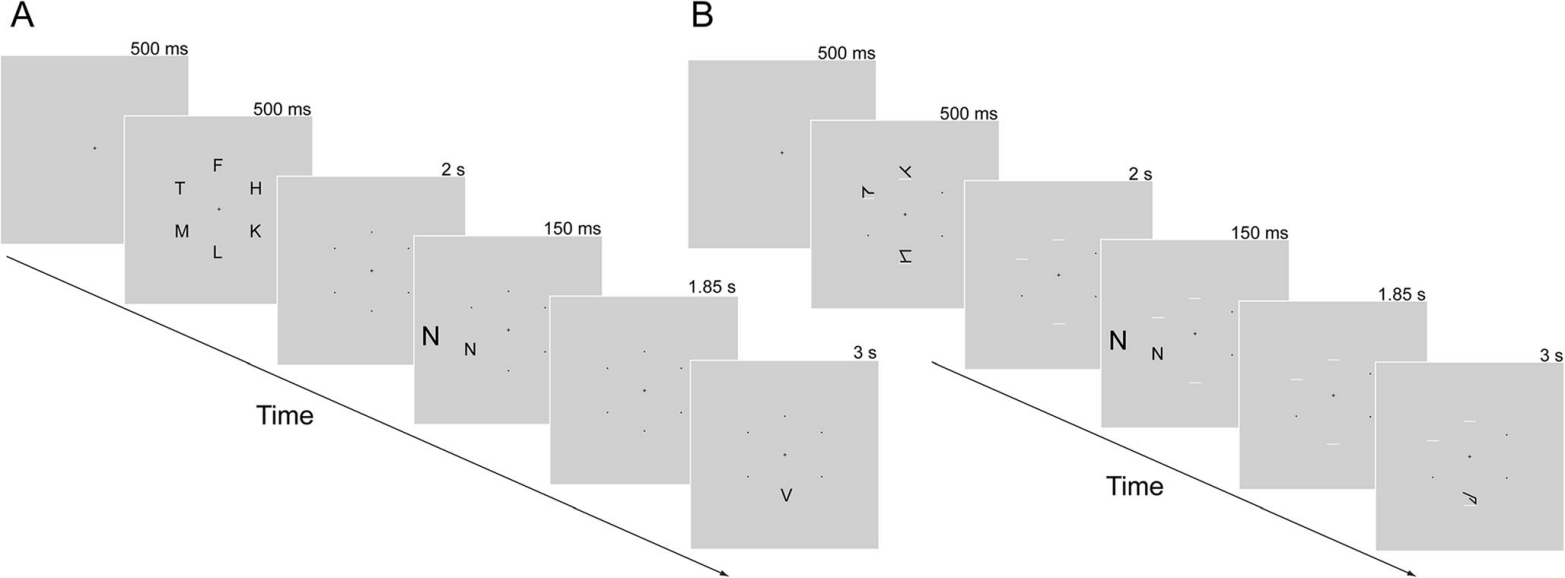
Experiment 2: Example trial sequence from the high memory load and incongruent distractor conditions in **a** Experiment 2a (working memory cognitive control load) and **b** Experiment 2B (visual short-term memory load). In the low-load conditions, only one item appeared in the memory set at the start of the trial. In the congruent condition, the target and distractor letters were the same. The correct memory probe response here is “different.” Display durations appear above each display. Stimuli are not drawn to scale

As was described earlier (in the introduction), increased load on cognitive control functions that serve to maintain stimulus processing priorities reduces their availability to maintain priorities in a selective attention task. Thus, loading WM cognitive control functions with the requirement to actively maintain other stimuli (e.g., rehearse a random set of digits) impairs the ability to control attention in accordance with stimulus-processing priorities, leading to increased processing of irrelevant distractors (e.g., De Fockert, Rees, Frith, & Lavie, 2001; Lavie, 2000; Lavie & De Fockert, 2005; Lavie et al., 2004). The aim of Experiment 2 was to replicate this effect (Experiment 2a) so that it could be compared with the effect of VSTM maintenance load (Experiment 2b). Figure 3 shows an example of the stimuli and procedure used in Experiment 2. Load was varied in both Experiments 2a and 2b by adding more items to the set. In Experiment 2a, the memory set items were letters that participants were requested to rehearse verbally. In Experiment 2b, the memory set items were meaningless symbols made from the same features of the letters used in Experiment 2a, while ensuring that none of the symbols resembled any particular letter, to discourage participants from verbalizing them (see Alvarez & Cavanagh, 2004, for a similar manipulation of VSTM load). Participants were instructed to maintain these symbols in visual memory by imagining them staying on the screen. As in Experiment 1, we predicted that increased VSTM load through higher set size would increase demands on visual representation capacity and, thus, lead to reduced distractor processing.

## Experiment 2a

### Method

#### Participants

Twelve new participants (mean age, 26.3 years; 7 women) took part in Experiment 2a.

#### Stimuli and procedure

Each trial started with a fixation cross presented at the center of the screen for 500 ms, followed by a 500-ms presentation of the memory set, which contained one (low load) or six (high load) letters (0.4° × 0.6°) randomly selected from F, H, K, L, M, T, V, W, Y, and X and placed with equal probability in any of six positions arranged in a circle of 2° in radius centered at fixation. In the low-load condition, small placeholder dots replaced five of the letters. Participants were instructed to verbally rehearse the memory set letters throughout the retention interval and to respond whether a memory probe letter appearing at the end of the interval was present or absent in the trial set.

The response competition task used in Experiment 1 (using target letters “N” and “Z” instead of the target letters “X” and “Z” in Experiment 1) was presented during the retention interval following a 2-s delay (in which a fixation cross and dots in each of the circle’s six positions were shown). A 2-s time window followed the response competition task, during which a speeded task response was made. A memory probe was then presented for 3 s (or until a response was made). The memory probe consisted of one letter equally likely to appear in any of the six locations in the high-load condition and always presented at the location of the memory set item in the low-load condition. On half of the trials, the memory probe’s identity matched that of the memory set stimulus. On the other half, the probe’s identity was equally likely to have been selected from the other letters in the memory set or from the remaining letters. Participants pressed one of two keys with their left hand, indicating whether the probe’s identity matched that of the memory set for that trial (“S” for same and “A” for different). Feedback in the form of the word “Wrong” presented for 500 ms at the center of the screen followed incorrect memory responses.

Load conditions were blocked. Following 64 practice trials (32 for each of the load conditions), each participant completed eight blocks of 64 trials each in ABBABAAB or BAABABBA order, counterbalanced across participants.

### Results and discussion

WM task accuracy rates were significantly lower in the high-load (*M* = 72 %, *SD* = 9 %) than in the low-load (*M* = 91 %, *SD* = 6 %) condition, *t*(11) = 8.122, *p* < .001, *d* = 0.76. WM estimates increased significantly from the low (*K* = 0.81, *SD* = 0.12) to the high (*K* = 1.93, *SD* = 1.16) WM load condition, *t*(11) = 3.49, *p* = .005, *d* = 1.13, indicating the significant draw on WM capacity as before.

Only data from correct WM task responses were entered into analyses of the response competition task. Trials with incorrect responses in the response competition task were removed from the RT analyses. A two-way repeated measures ANOVA on the response competition task RTs with the factors of WM load (low, high) and distractor congruency (congruent, incongruent) revealed a main effect of congruency, *F*(1, 11) = 12.12, *p* = .005, *η* ^2^ = .52, indicating longer RTs in the presence of incongruent versus congruent distractors (Fig. 2). There was no main effect of WM load (low load, *M* = 513 ms, *SD* = 122 ms; high load, *M* = 525 ms, *SD* = 114 ms), *F*(1, 11) = 1.90, *p* = .20, *η* ^2^ = .15. Importantly, distractor congruency effects were increased with higher cognitive control WM load, as indicated by the significant interaction, *F*(1, 11) = 8.72, *p* = .013, *η* ^2^ = .44, as we predicted. There were no differences in the arcsine-transformed accuracy rates between the different conditions (all *F*s < 1). The effect of load on distractor congruency was driven mostly by high WM load increasing interference caused by the incongruent distractor (low load, *M* = 532 ms, *SD* = 134 ms; high load, *M* = 556 ms, *SD* = 121 ms), *t*(11) = 2.25, *p* < .05, *d* = 0.19), rather than a suppression of the congruent distractor (low load, *M* = 495 ms, *SD* = 115 ms; high load, *M* = 493 ms, *SD* = 111 ms), *t* < 1.

These results replicate previous findings (De Fockert et al., 2001; Lavie, 2000; Lavie & De Fockert, 2005; Lavie et al., 2004; Rissman, Gazzaley, & D’Esposito, 2009) and demonstrate that when cognitive control WM processes are occupied, people are more susceptible to interference by irrelevant distraction.

## Experiment 2b

### Method

#### Participants

Seventeen new participants (mean age, 25.8 years; 12 women) took part in Experiment 2b.

#### Stimuli and procedure

Pilot testing using the same stimuli and procedure but a VSTM set size of six items revealed that memory performance was close to chance in the high VSTM load condition (*M* = 53 %, *SD* = 3 %). Therefore, in order to match performance to the cognitive control WM load (Experiment 2a) and the previous VSTM load manipulation, we used a memory set size of three items in the high VSTM load condition.

The memory set stimuli thus consisted of one (low load) or three (high load) meaningless symbols that were randomly drawn from a pool of 500 different stimuli. These symbols were generated with an algorithm in MATLAB that created meaningless symbols based on basic features of the letters used. For each symbol, the number of features was matched to the average number of bars used for each letter in Experiment 2a. All symbols generated were individually screened, and any letters that resembled English letters were excluded from the pool of stimuli.

Participants were instructed to maintain these symbols in visual memory by imagining them staying on the screen throughout each trial. Three placeholders in the form of horizontal bars were displayed under the locations of the memory items in the high-load condition or under the single memory item location and two other randomly selected locations in the low-load condition. The placeholders remained visible for the entire trial period to aid visual maintenance of the memory set items by projecting them at the locations indicated during the delay. The target letter in the selective attention task never appeared in a location with a placeholder under both conditions of load, so that spatial uncertainty was matched across load conditions.

### Results and discussion

VSTM task accuracy was reduced from the low (*M* = 86 %, *SD* = 9 %) to the high (*M* = 63 %, *SD* = 10 %) VSTM maintenance load condition, *t*(16) = 13.870, *p* < .01, *d* = 0.86. VSTM capacity estimates increased significantly from the low (*K* = 0.72, *SD* = 0.18) to the high (*K* = 1.58, *SD* = 1.15) VSTM load condition, *t*(16) = 3.45, *p* = .003, *d* = 0.94, indicating once again a significant draw on VSTM capacity with higher load. Mixed-model ANOVAs comparing the arcsine transformed accuracy rates and memory capacity estimates (*K*) as a function of load between Experiments 2a and 2b showed no effect for experiment, for accuracy rates, *F*(1, 27) = 1.91, *p* = .18, η^2^ = .07, and for *K* capacity estimates, *F* < 1, and importantly, no interaction (both *F*s < 1). Thus, the effect of load on memory capacity was comparable between the different manipulations of cognitive control WM load and VSTM maintenance load.

A two-way repeated measures ANOVA of the response competition task RT with the factors of VSTM load (low, high) and congruency (congruent, incongruent) revealed a main effect of congruency on RTs, *F*(1, 16) = 76.956, *p* < .001, *η* ^2^ = .83, and no main effect of VSTM maintenance load (low load, *M* = 679 ms, *SD* = 183 ms; high load, *M* = 666 ms, *SD* = 169 ms), *F*(1, 16) = 1.809, *p* = .197, *η* ^2^ = .10, as before. Importantly, a significant interaction between VSTM maintenance load and distractor congruency, *F*(1, 15) = 5.922, *p* = .027, *η* ^2^ = .27, indicated a reduced distractor congruency effect with high, as compared with low, VSTM maintenance load (see Note 3). Once again, a closer inspection of the congruency data suggested that the finding was mostly due to high load reducing interference by the incongruent distractor (low load, *M* = 713 ms, *SD* = 191 ms; high load, *M* = 691 ms, *SD* = 176 ms), *t*(16) = 2.00, p = .063, *d* = 0.12, rather than facilitation by the congruent distractor (low load, *M* = 645 ms, *SD* = 175 ms; high load, *M* = 641 ms, *SD* = 163 ms), *t* < 1.

A similar repeated measures ANOVA on the arcsine-transformed response competition task accuracy rates revealed a significant main effect of congruency, *F*(1, 16) = 5.90, *p* = .03, *η* ^2^ = .27, no main effect of VSTM load (low load, *M* = 1.31, *SD* = 0.12; high load, *M* = 1.29, *SD* = 0.16), *F*(1, 16) = 1.46, *p* = .245, *η* ^2^ = .08, and no interaction, *F* < 1.

The opposite effects on response competition during the memory delay found for cognitive control WM load (Experiment 2a) versus VSTM load (Experiment 2b) were further confirmed in the finding of a significant interaction between load level and load type in a mixed-model ANOVA conducted on the distractor RT congruency effects using the within-subjects factor of load level (low, high) and the between-subjects factor of load type (cognitive control or visual maintenance), *F*(1, 27) = 14.64, *p* = .001, *η* ^*2*^ = .35.

We note, however, that there was a main effect of experiment in this ANOVA, indicating that, overall, RT was longer in Experiment 2b than in Experiment 2a.^4^ Since overall, RT was also longer in Experiment 1b (which also manipulated VSTM load, as compared with Experiment 1a [see Note 3]), this seems to suggest that the addition of visual maintenance (whether high load or low load) during performance of a response competition task slows down performance more than does either verbal maintenance (Experiment 2a) or encoding (Experiment 1a). This may indicate a greater difficulty in dual-task coordination between response competition and visual, as compared with verbal, maintenance, as well as compared with encoding.

Importantly, the effects of the different types of load were both established within each task and are, thus, unaffected by the potential difference in overall task coordination between the different tasks.

Furthermore, a mixed-model ANOVA comparison of the distractor RT congruency effects between Experiments 1b and 2b and the within-subjects factor of load (low, high) confirmed the similar pattern of results in Experiments 1b and 2b, *F* < 1.

## Individual-differences analysis: trade-off between the VSTM load effect on capacity estimates and on distractor response competition effects

**Fig. 4:**
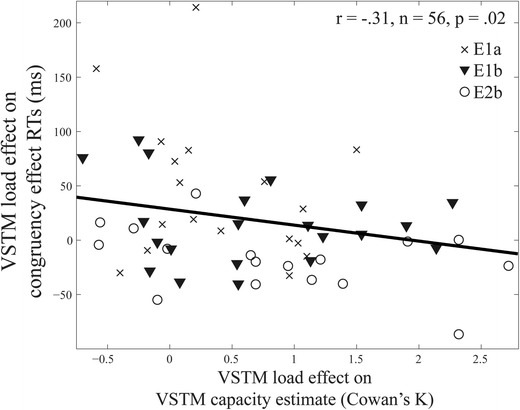
Scatterplot with individual data points from the three experiments that employed a visual short-term memory (VSTM) task illustrating the relationship between the VSTM load effect on the congruency effect response times (RTs; *y* axis) and on VSTM capacity estimates (*x* axis; Cowan’s *K*). The line represents the least square linear fit

To further investigate the effects of VSTM maintenance load on distractor processing, we pooled together data from 56 participants^5^ from the three experiments that used a VSTM task with similar methods: Experiment 1a (VSTM encoding load), Experiment 1b (VSTM maintenance load using colored squares), and Experiment 2b (VSTM maintenance load using meaningless shapes). Using Pearson product–moment correlation analysis, a significant negative correlation was found between the effect of VSTM load on the VSTM capacity estimate (i.e., the difference in Cowan’s *K* between low- and high-load conditions) and on the response competition effect RT (see Fig. 4), *r* = −.31, *n* = 56, *p* = .02. This result provides further support for our claim that the level of distractor processing depends on the extent to which VSTM resources are occupied.

## General discussion

The present research demonstrates dissociable effects of different types of WM load on selective attention as measured by distractor interference effects in the well-established response competition task (Eriksen & Eriksen, 1974; Lavie, 1995). Load on visual representation resources required for visual maintenance or encoding of color and location (rce interference by an irrelevant distractor in the response competition task presented either during encoding of the set or during the maintenance delay. These effects are similar to the effects of perceptual load (Lavie, 1995) and are in support of our hypothesis that visual perception, encoding, and visual maintenance tax common sensory visual representation resources (see also Pasternak & Greenlee, 2005; Serences et al., 2009). Additional support for this conclusion was provided by a negative correlation between the effect of load on individual VSTM capacity estimates and on distractor interference (see individual-differences analysis). This demonstration of a trade-off between the extent to which VSTM resources were occupied in high load and the extent to which distractor interference effect was reduced with higher load suggests shared resources between visual perception and VSTM, as we predicted. Interestingly, this correlation also suggests that individuals for whom capacity estimates were not increased much under higher load (in other words, those with lower capacity estimates in high load) were more prone to distractor interference. This observation is consistent with previous demonstrations that evoked potential responses to the distractor stimuli during VSTM maintenance are negatively correlated with VSTM capacity, so that individuals with a lower VSTM capacity are more likely to process irrelevant distractors (Vogel, McCollough, & Machizawa, 2005).

In contrast, distractor interference in the response competition task was increased when it was presented during the memory delay of a verbal WM task under a higher load that required active rehearsal, a function that has long been associated with cognitive control WM load, consistent with previous findings (De Fockert et al., 2001; Lavie et al., 2004).

The opposite effects of different types of memory load rule out alternative accounts for these results in terms of a general increase in task difficulty. Although higher load increased the overall task difficulty to an equivalent level across the different load manipulations, critically the effect on distractor interference depended on whether visual representations (both during encoding and during maintenance) or cognitive control WM processes were loaded.

These findings provide a new line of support for the dissociation between WM functions of storage and cognitive control (e.g., Baddeley, 1996; Repovs & Baddeley, 2006; Smith & Jonides, 1999). Previous evidence in support of this dissociation comes mainly from the different patterns of cortical activations found for tasks that use storage versus cognitive control processes (e.g., D’Esposito et al., 1995; Miller & Cohen, 2001), as well as neuropsychological reports of patients that show deficits in storage but not in cognitive control processes and vice versa (e.g., Baddeley, 2012; D’Esposito & Postle, 2000). Our demonstration that distraction can be either reduced or increased depending on whether load is increased on WM maintenance or cognitive control provides new behavioral evidence for the dissociation of these WM functions.

Our findings extend load theory to accommodate the effects of different types of WM load on selective attention. The findings demonstrate the importance of careful consideration of the exact mental process that is loaded. Indeed, this demonstration that load on cognitive control WM processes has an opposite effect on selective attention to load on visual maintenance processes can account for some apparent discrepancies in the previous literature. Much previous research has shown that distractor processing is increased with high WM cognitive control load, using manipulations similar to those used here (e.g., Carmel, Fairnie, & Lavie, 2012; De Fockert et al., 2001; Lavie, 2000; Lavie & De Fockert, 2005; Lavie et al., 2004; Rissman et al., 2009), but other studies have reported reduced distractor processing with high WM load (Bollinger, Masangkay, Zanto, & Gazzaley, 2009; Roper & Vecera, 2014; Rose, Schmid, Winzen, Sommer, & Buchel, 2005; Sreenivasan & Jha, 2007). While these opposite effects may appear contradictory under a unitary WM concept, they are explained by our proposed dissociation. The reports of reduced distractor interference with higher load have been obtained in VSTM tasks that require maintenance of either one image (low load) or several images (higher VSTM load), similar to our manipulation of VSTM set size, during visual distractor (e.g., faces) processing. These findings are consistent with the present research. Overall, our view states that distractor processing can be reduced or increased depending on the WM process that is loaded, visual maintenance or cognitive control. Moreover, as we state earlier, our findings of opposite effects of load in WM cognitive control load versus load in VSTM maintenance, despite equivalent increase in task difficulty, allow us to clearly attribute the effects of each manipulation to the specific draw on either maintenance or control capacities, whereas in the lack of such control, each of the previous research findings, when taken in isolation, remains open to alternative accounts in terms of a general increase in the general task difficulty.

Although the focus of this work was on establishing the new effect of VSTM load on distraction, it is perhaps worth noting that demands on cognitive control can also be increased by the requirement to coordinate dual tasks, and since dual-task coordination taxes control over stimulus-processing priorities, distractor effects are found to increase in dual- versus single-task conditions (see Lavie et al., 2004, Experiments 4 and 5). Indeed, the effects of dual- versus single-task coordination do not require that the added task will involve a high WM load or a WM task at all. For instance, Brand-D’Abrescia and Lavie (2008) found that distractor response competition effects were increased in dual-task, as compared with single-task, conditions, when the dual-task conditions involved auditory pitch discrimination or visual line discrimination tasks. Lavie et al. (2004) demonstrated that the very same conditions of low WM load in their Experiments 1–3 led to increased distractor effects when used in dual-task conditions, as compared with a single-task condition, and Burnham, Sabia, and Langan (2014) using a similar dual- versus single-task comparison found that both low- and high-load conditions of a WM task led to increased attention capture by a color singleton in the dual- (vs. single-) task conditions. Interestingly, Burnham et al. found these effects not just with the use of executive control WM tasks (such as backward counting), but also with VSTM tasks. However, since the level of VSTM load had no effect on distraction and this varied only as a function of demands on dual- (vs. single-) task coordination, these results can be clearly accommodated within the load theory proposals regarding the role of cognitive control load in distractor interference. Another line of work has emphasized the importance of considering whether WM load selectively affects either the processing of the target or the processing of the distractor in response competition tasks. This research demonstrated that WM tasks that selectively draw on resources involved in either the target (but not distractor) processing or vice versa led to increased processing of the stimulus that was not loaded (e.g., increased distractor processing when WM selectively loaded on target processing; Kim, Kim, & Chun, 2005; Park, Kim, & Chun, 2007). The finding that distractor processing is reduced when the WM load manipulation shares resources with processing of the distractor is generally consistent with our present results (concerning the effects of VSTM load). However, in our selective attention task, the same stimuli were used for both target and distractors (both were visually presented letters); thus, the effects of either of the load manipulations that we employed cannot be due to a selective effect on either target or distractor processing (cf. Kim et al., 2005; Park et al., 2007).

Indeed, our results pattern established a selective effect of memory load on the distractor response competition effects, with no effects on the target RTs. It appears that similar to previous research, our use of a response competition task of low perceptual load with just one target letter is likely to have rendered it less sensitive to the effects of load on overall RT and more sensitive to reveal effects on the processing of the peripheral distractors. This result is consistent with previous findings. For example, Lavie and De Fockert, (2005) and Burnham et al. (2014) also used a distractor task of low load (a color singleton pop-out search) and found effects of increased distraction in the absence of effects on the overall search RTs. De Fockert et al.’s (2001) behavioral results showed a similar pattern, and so did Lavie et al.’s (2004, Experiments 1, 3). In the present study, the selective effects of load on distractor processing suggest that any visual representation capacity not taken up by the VSTM task under conditions of high VSTM load was sufficient for detection of the single target item (which is why target detection RT remained unaffected), but not for the perception of the irrelevant peripheral distractor letter to the level that can produce a robust response competition effect (as evidenced by the reduced response competition effects with higher VSTM load).

An important question that arises is what is the source of distractor processing modulation. Konstantinou et al. (2012) have recently shown that high VSTM load reduces both retinotopic visual cortex response to contrast and detection sensitivity (measured with *d′*) during the memory delay. Furthermore, Konstantinou and Lavie (2013) have shown that when participants performed a visual search task (somewhat similar to that used here, but with no distractor) during the delay and detection was measured for a low-priority search-irrelevant stimulus in the periphery, VSTM load and cognitive control WM load had opposite effects on detection. Whereas VSTM load reduced detection sensitivity, cognitive control WM load increased it. These findings suggest that the modulation of the distractor response competition effects established here are due to reduced perceptual processing of the distractor letters. These suggestions are consistent both with the claim that VSTM shares sensory representations with perception (e.g., Pasternak & Greenlee, 2005) and with previous findings that cognitive control WM load can enhance distractor perception both in the inattentional blindness paradigm (De Fockert & Bremner, 2011) and in the two-alternative forced choice recognition paradigm (Carmel et al., 2012).

In conclusion, the present findings enhance our understanding of how WM and selective attention interact. WM load can be either detrimental or beneficial to focused attention, depending on whether maintenance or cognitive control functions are loaded. Future research may determine whether these effects can extend to other measures of distraction—for example, attentional capture by singleton items during search (e.g., Lavie & De Fockert, 2005; Theeuwes & Burger, 1998) or attentional capture by entirely irrelevant distractors (e.g., Forster & Lavie, 2008a, 2008b)—and to loading maintenance in other modalities. For example, Dalton, Lavie, and Spence (2009a) and Dalton, Santangelo, and Spence (2009b) demonstrated that both auditory and tactile distraction is increased with high cognitive control WM load. Would both tactile and auditory distraction be reduced with high maintenance load in the auditory and tactile modalities, respectively? Our research and potential future directions emphasize the importance of considering not only the level of load in the immediate visual environment, but also the level and type of load in WM in order to understand and predict people’s ability to focus attention.

## Footnotes

1 We note that our response competition task involved low perceptual load with only one target letter that should have been detected rapidly in a “pop-out” manner from among the dot place holders. This suggests that target processing would be conducted during encoding of the memory set items in Experiment 1a, rather than being deferred to the maintenance stage during the delay.

2 To examine whether the spatial distance between the target and distractor letters had any effect, we conducted an additional ANOVA on the letter search task RTs, with the factors of WM load (low, high), congruency (congruent, incongruent), and distractor-to-target distance (close, far). This ANOVA revealed no main effect of or interaction with distractor to target, *F* < 1. These results rule out alternative accounts for our load effects in terms of any spatial interactions.

3 Examination of the response competition RT data suggests that target RTs were longer, overall, in Experiment 1b, as compared with Experiment 1a, *F*(1, 38) = 17.54, *p* < .001, *η* ^2^ = .32, and the overall magnitude of distractor effects was larger (1a, *M* = 48 ms, *SD* = 57 ms; 1b, *M* = 118 ms, *SD* = 100 ms), *F*(1, 38) = 6.75, *p* = .01, *η* ^2^ = .15. The RT difference was found already in the low-load conditions, in which the task difference in demand was minimal (e.g., the encoding of the single letter and single colored square should have occurred in a speedy parallel manner, whether they are presented together within the same display [Experiment 1a] or apart [Experiment 1b]). Thus, the use of two different participant groups was a more likely cause for the overall RT difference. Indeed, a close inspection of the data revealed 6 participants with a response competition task RT of more than 2 *SD*s longer (*M* = 1,044 ms) than the group mean (*M* = 669 ms, *SD* = 171) in the low-load condition of Experiment 1b. A reanalysis of the search data following exclusion of these participants did not change the results pattern or significance. Thus, the VSTM load effect on congruency did not depend on the speed of the overall search RT. We note, however, that even with the exclusion of the outlier participants, the overall RT remained longer in Experiment 1b (*M* = 650 ms, *SD* = 150 ms) than in Experiment 1a (*M* = 487 ms, *SD* = 162 ms), *F*(1, 32) = 9.32, *p* = .005, *η* ^2^ = .27. The overall magnitude of distractor effect also remained higher in Experiment 1b (*M* = 117 ms, *SD* = 110 ms) than in Experiment 1a (*M* = 48, *SD* = 57), *F*(1, 32) = 5.26, *p* = .02, *η* ^2^ = .14 (even remained larger when expressed as percentage of the RT: *M* = 18 % vs. *M* = 10 %, respectively). This perhaps points to greater overall demands on task coordination and cognitive control involved in the task used in Experiment 1b, as compared with Experiment 1a. Importantly, a larger distractor effect overall (across the load conditions) in Experiment 1b was found also in comparison with Experiment 2b, which also varied VSTM load, *F*(1, 38) = 5.62, *p* = .02, *η* ^2^ = .13. Thus, the cause for greater overall distraction was specific to that experiment and does not reflect any general effect of VSTM maintenance tasks on distraction.

4 Examination of the data in Experiment 2b showed that the response competition target RT appeared longer than in Experiment 2a. Close inspection of the data did not reveal any outliers. We therefore conducted a median split analysis of the data according to the mean overall RT. The long RT group (*M* = 900 ms, *SD* = 184 ms) and short RT group (*M* = 554 ms, *SD* = 86) both showed the same pattern as the overall results (if anything, the effect of load on congruency was numerically larger for the short RT group). Thus, the VSTM load effect on distractor processing did not depend on the target RT.

5 The data of 1 outlier participant from Experiment 1b with a distractor response competition effect of more than 2 *SDs* above the mean were excluded from the analysis.
